# Return to Performance of a Soccer Player with an Adductor Longus Injury: A Case Report

**DOI:** 10.3390/medicina60121998

**Published:** 2024-12-03

**Authors:** José Luis Estévez Rodríguez, Jesús Rivilla García, Sergio Jiménez-Rubio

**Affiliations:** 1 Switzerland National Team, Worbstrasse 48, 3074 Muri bei Bern, Switzerland; 2 Facultad de Ciencias de la Actividad Física y del Deporte (INEF-Sports Department), Universidad Politécnica de Madrid, 28040 Madrid, Spain; jesus.rivilla.garcia@gmail.com; 3 Sports Science Research Studies, Universidad Rey Juan Carlos, 28943 Fuenlabrada, Spain; sjimenezrubio@yahoo.es; 4 Research Department Train Movements Center, 28922 Alcorcón, Spain

**Keywords:** return to play, rehabilitation, GPS, groin injuries, reconditioning

## Abstract

*Context:* There is limited information on the quantification of external load and reconditioning programs during adductor longus injuries in soccer. *Case Presentation:* This case report describes a male professional soccer player (*LaLiga*) returning to performance following an adductor longus muscle injury during the 2022/2023 season. The player suffered the injury during a change of direction in a match. The injury was confirmed by ultrasound after 48 h, and the previously validated rehabilitation and reconditioning program was applied to the injured player. This case report has focused on the development of the on-field reconditioning program and the quantification of the load during this phase. The goal of this case report was to return the player to pre-injury loads using global positioning systems (GPS). Variables such as total distance, distances covered at different speeds and metabolic load variables were quantified during the injury process, with the aim of increasing them through training and reaching at least 75% of the game load. Therefore, objective performance criteria for making return-to-play decisions based on the use of GPS was determined. In addition, the return to play (RTP) was on the 20th day after the injury, and then four RTPs were recorded in the following 6 weeks after the injury occurred, without re-injury. *Conclusions:* The approach to the competition performance profile, through the quantification of the external load during the rehabilitation process of the injured player, allowed us a safe return to competition and continued competition with a 6-week follow-up.

## 1. Introduction

Soccer is a complex sport in which a multitude of tactical, technical and physical elements modulate performance [1]. Knowing the locomotor and physiological demands will be one of the keys to a better understanding of a player’s positional and match requirements [2,3]. The use of global positioning system (GPS) technology both in training and in official matches is an effective tool to control the accumulated load of players [3]. The main objective of adapting the physical demands of the game to training is to ensure that players receive an optimal physical stimulus in relation to the competition and positional demands to maximize their physical performance and reduce the risk of injury [1]. A player’s tactical role appears to be a key determinant of his physical performance in the match, so it is imperative that the conditioning stimulus has a positional element [4]. Nowadays, one of the biggest problems limiting the optimal development of an athlete’s performance is injuries. Specifically, muscle injuries continue to be a major challenge for club, medical and technical staff [4,5]. The incidence of injuries in soccer shows that most of them have a significant impact on the lower limb. Injuries affecting the adductor muscle are among the most frequent, accounting for 24% of all muscle injuries and the second highest incidence group [5]. Moreover, recent studies show that the mean duration of absence is 21 days [6].

Groin pain provides a massive challenge for all those involved in the diagnostic, rehabilitation and physical preparation of athletes at all levels due to the complex anatomy of the groin region and the poor understanding of the adverse mechanisms that predispose the athlete to injury [7]. Intradisciplinary work and the combination of information from each of the parties will help technical and medical staff to make better decisions during the return to play [8]. A recent study, based on a Delphi questionnaire, indicated that strength assessment, performance testing analysis and sport-specific skills assessment can be considered useful in the evaluation of return to play in athletes with long-standing adductor-related groin pain [9].

Currently, there are numerous studies [1,2,10,11] in which GPS technology has been used to quantify and manage load during training, matches and also with injured athletes after return to play. Some of these studies have defined the intervention program carried out with the injured athlete, focusing on indoor and on-field training during the injury. But, there are a limited number of studies [12] or case reports showing the use of GPS during the injury process, specifically in injuries involving the adductor longus. The use of GPS during the training of the injured athlete can help us to improve rehabilitation processes, providing a better intervention for injuries and consequently improving the results in terms of performance and reducing the risk of injury. Considering the quantification of the load during on-field training can be a key performance criterion to improve decision-making in the process of return to training and return to play. The goal of this case report was to describe the rehabilitation and reconditioning process of a player with an adductor longus injury, focusing on the return to training to pre-injury loads using global positioning systems.

## 2. Case Presentation

A 26-year-old male professional soccer (Guinea-Equatorial) with a previous history of muscle injuries, mainly in the hamstrings and rectus femoris in both lower limbs. He is a predominantly left-footed player and has an average body mass of 71 kg and a height of 173 cm. The player had a history of having played 250 professional matches as a midfielder. This player competed in the Second Division of professional soccer (*Liga SmartBank*) of the Spanish soccer league system, commonly known as *LaLiga*, during the 2022–2023 season. The player provided written informed consent, and the procedures were conducted in accordance with the Declaration of Helsinki. The study was approved by the ethical committee of the Universidad Rey Juan Carlos (nº registration: 1601202303423; 9 March 2023).

During the match, around minute 70 of the second half, while the player was performing a change in direction, he felt a discomfort in his left adductor. He continued to compete with the discomfort for at least 10 more minutes; the pain and discomfort increased until he was finally substituted. At the end of the match, the physical therapist performed clinical pain provocation tests, which have previously been published [13]. The physical therapist examined the passive range of motion in a side-lying hip abduction position and performed the bent knee fall-out test, as well as assessed eccentric strength in side-lying hip adduction and side-lying hip abduction positions. The player showed pain during maximum contraction. On the other hand, the player did not feel any discomfort during stretching tests. After the first examinations, 48 h later, an ultrasound scan confirmed a muscle injury in the adductor longus, very close to the myotendinous junction (MTJ), and classified it as a grade 1–2 muscle injury [14].

**Figure 1:**
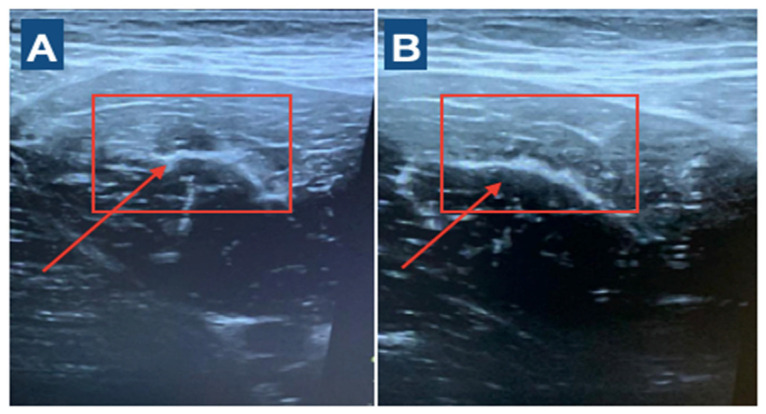
(**A**). Initial 48 h post-injury. Ultrasound imaging shows muscle fiber discontinuity at the level of tear. (**B**) Ultrasound imaging at 9 days post-PNE.

The player was clinically diagnosed by ultrasound [15] and subsequently underwent invasive physiotherapy treatment with percutaneous musculoskeletal electrolysis (PNE) with a dry needling needle. The decision to use this technique is supported by the club’s medical staff, the results obtained from previous injuries and the wide experience in the use of this technique. In addition, we have previously shown its efficacy in muscle injuries [11]. Electrolysis was performed 48 h after injury under ultrasound control and ultrasound-guided treatment at an intensity of 2 mA for 3 s and repeated five times [11] (Figure 1). A physiotherapist with more than 5 years of experience in ultrasound evaluation and over 7 years of experience in invasive therapy applied the PNE technique. The rehabilitation and reconditioning program started at 24 h post-PNE. It was based on a previously validated program that we have developed for adductor longus injury [10,11]. In addition, the tissue repair process was assessed using an ultrasound scan every 4–5 days to evaluate the evolution of the process and to be able to manage the subsequent loads that were applied to the injured tissue.

**Figure 2:**
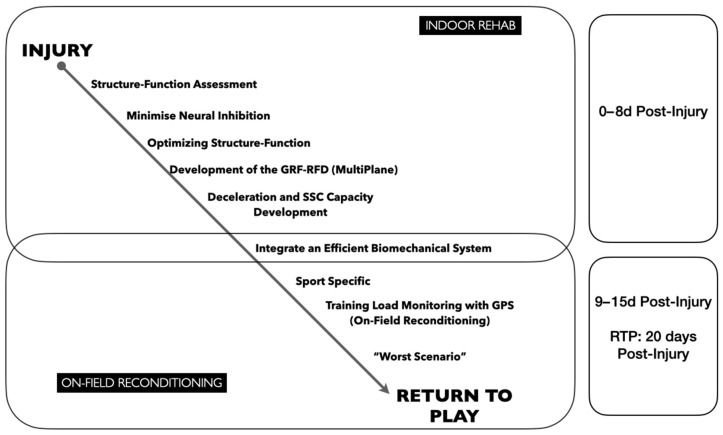
Rehabilitation and reconditioning program. The figure shows the contents of the indoor and on-field programs, which were developed from the time of injury until return to play. GRF: ground reaction force; RFD: ratio force development; SSC: stretch shortening cycle; RTP: return to play.

The program lasted 15 days and was divided into two parts (Figure 2). The first part lasted 8 days from the moment of the injury until the end of this phase and the start of the reconditioning phase on the field, which lasted 7 days. In this case report, we will focus on defining the work conducted in the reconditioning phase, with special emphasis on the quantification of loads through GPS in order to obtain values closer to those of the pre-injury competition.

The first phase of the rehabilitation process was mainly based on the assessment and intervention of the injured structure. In addition, this phase was carried out to develop the ground reaction force (GRF), and one of the objectives was to improve the deceleration capacity and the development of a plyometric program to prepare the structure for later demands, which will take place during the second phase of the program. These training contents are fully developed in the specific program [10,11] that we have applied to the injured player.

**Figure 3:**
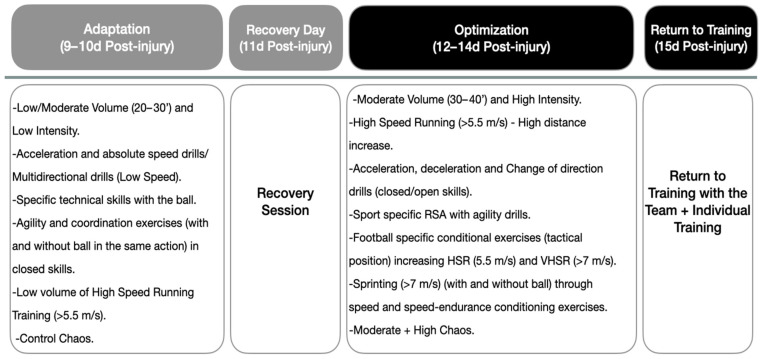
On-field reconditioning program. The figure shows the training contents that were developed during the on-field phase. RSA: repeated sprint ability.

During the on-field reconditioning program, the first phase was called the adaptation phase (Figure 3), which was based on the development of low-intensity and low-volume work on the pitch, and this phase was also characterized by the return to running, as well as deceleration, acceleration and change in direction actions developed at low speed. Exercise selection and programming were based on the optimal loading concept to maximize the physiological adaptation of the involved structures [12]. The second part of the program, called optimization (Figure 3), will last three sessions, and we will try to increase the values of the variables related to high intensity, trying to achieve values closer to the injured player’s competition profile.

**Figure 4:**
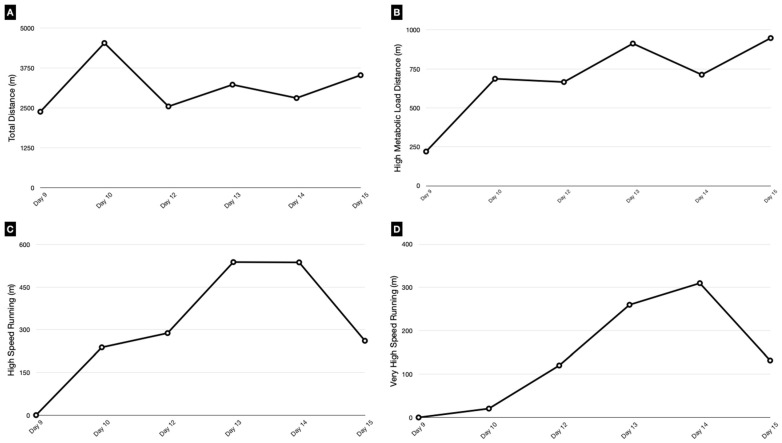
Control load (GPS) on-field reconditioning program. The graph shows the quantification of loads during the on-field phase. It represents the different variables that were measured ((**A**) Total Distance; (**B**) High Metabolic Load Distance; (**C**) High-Speed Running; (**D**) Very High-Speed Running).

Throughout this phase, we increased total distance, manipulating exercise intensity and high metabolic load distances by altering the work/rest ratio between intermittent efforts. The goal during this phase was to expose the player to submaximal running speeds (>75% High-Speed Running (HSR) and Very High-Speed Running (VHSR) pre-injury). Training performance during on-field reconditioning was recorded using GPS accelerometer units (WIMU PRO™, Real Track Systems^®^, Almeria, Spain). The GPS devices obtained data with a frequency of 18 Hz by GPS Galileo. The variables used to record performance were (a) total distance (TD), (b) High-Speed Running (HSR) distance (>5.83 m/s–>21 km/h), (c) Very High-Speed Running (VHSR) distance (>6.67 m/s–>24 km/h), and (d) High Metabolic Load Distance (HMLD) (distance greater than 25.5 W kg^−1^). Previous studies have used these GPS metrics to quantify performance in injured players [10,11,12,16,17]. For this purpose, we used the pre-injury values of these variables as a reference for planning the running load targets (Figure 4).

The player was instructed to perform the included training within a pain score of 2 on a numerical rating scale from 0 to 10, where 0 represented no pain, and 10 was the most extreme pain possible. The player was encouraged to increase the load with minor pain corresponding to 2 of 10; that is, if the pain was ≥3 of 10, the load was reduced. The program was implemented and supervised by a rehabilitation physical coach with individual sessions. Decisions regarding training load were made by medical and technical staff, taking into account GPS data and the player’s pre- and post-training subjective feedback.

**Figure 5:**
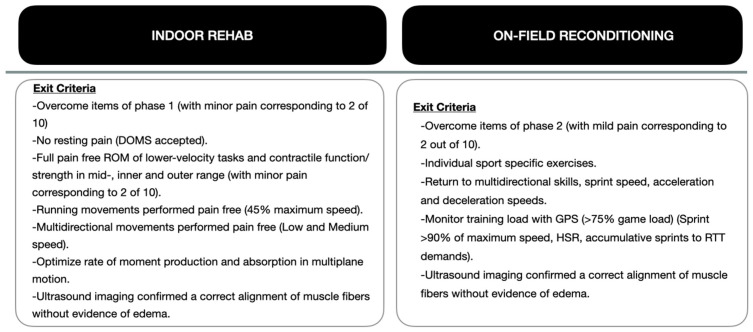
Exit criteria for each phase of the program. DOMS: delayed onset muscle soreness; ROM: range of motion; RTT: return to training.

When the player successfully completed all points of the program and passed the progression criteria, he was declared fit to train with the team. To achieve this, we relied on different exit criteria for each phase of the rehabilitation and reconditioning program (Figure 5) that were based on a combination of objective injury-relevant and training load data, ultrasound scan results highlighting healing and maturation and positive feedback/lack of pain reported by the player. One of the criteria we took into account was the training load measured with GPS, reaching the different variables for 75% of the game load; these criteria have already been defined by previous studies [10,11,12,17]. Finally, we concluded that the player’s level of risk tolerance was above the estimation of reinjury risk, and 15 days post-injury, the player resumed team training.

The return to training (RTT) with the team was on the 15th day of injury, where the player participated in a part of the training with the team. This consisted of a warm-up and participation in small sided games (SSGs), plus an extra individual session in the field for an approximate volume of 20 min; it had similar content to the last days of the reconditioning training program, as shown in Figure 3. The return to competition was on the 20th day of injury; from then on, we observed five RTPs, which correspond to the post-injury follow-up.

**Figure 6:**
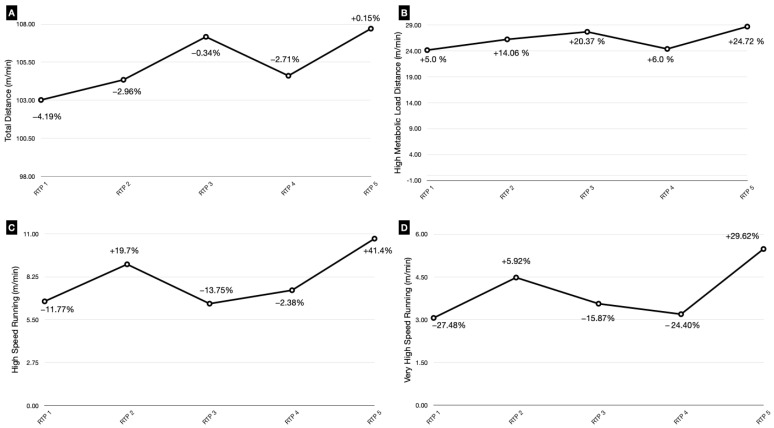
GPS match data during return to play. The graph shows the competition profile of the injured after RTP and compares it with the pre-injury competition profile. In it are represented the different variables that were measured ((**A**) Total Distance, (**B**) High Metabolic Load Distance, (**C**) High-Speed Running, and (**D**) Very High-Speed Running). RTP: return to play.

It should be noted that we created a competition profile of the player before the injury, where we chose the last five matches before the injury, in which the player had played at least 75 min, and we obtained an average performance profile. We consider that selecting the last game prior to the injury may be insufficient. In addition, there are important variations in data between matches that are related to multiple factors, such as technical decisions, game model, tactical aspects, number of substitutions and expulsions. This profile was used to compare the data obtained during the on-field phase in order to approach the values obtained in competition. In addition, Figure 6 shows a comparison of the GPS match data of the different variables that were measured, where the player’s performance and comparison with the pre-injury profile can be observed.

## 3. Discussion

This case report presents the quantification of load using GPS during the reconditioning process after an adductor longus injury. The aim of the report was to return the player to pre-injury loads. On the other hand, performance parameters were registered after the return to play to assess the effectiveness of the intervention and the effects on the detraining caused by the injury.

Previous studies have reported an injury absence for adductor longus injury of about 21 days on average [6]. The results indicated that the intervention program was successful, as layoff duration was reduced, allowing the return to training on injury day 15. Like other recent studies, it justifies the importance of early intervention on the injured tissue to favor the repair process [16,17]. The duration of the return to training was 15 days, and the return to play was 20 days. The player returned to play earlier and did not suffer any relapses during the follow-up period. The layoff duration is an accurate tool to assess the recovery of the athlete since it does not depend on an exact date of competition; rather, it depends on the capacity of the player to overcome loads applied during reconditioning. Moreover, the results related to layoff duration were similar to those obtained in the program applied by Jiménez Rubio et al. (2021), which confirms the effectiveness of the program. Multidisciplinary and interdisciplinary work will allow us to achieve the objectives related to injury risk reduction, providing the player with optimal care through integrated performance and health management. Good communication between the performance and sports medicine teams facilitated the shared decision-making process, facilitating the player’s return to training with the group and to competition.

Previous studies have used performance variables as the most relevant for measuring performance in soccer players after return from injury [10,11,12,17]. In addition, these have been considered useful in the evaluation of RTP in athletes with long-standing adductor-related groin pain [9]. This case series should be interpreted with caution. One of the limitations of our study was not including variables such as maximum speed, maximum acceleration and maximum decelerations, which other authors have used in their studies [10,11,12,16,17] to measure the performance of healthy and injured athletes. Maybe these are variables that we should manage in the process of rehabilitation and RTP since variables such as acceleration and deceleration are intimately related to the change in direction, one of the main mechanisms of adductor injuries, so it will be interesting to quantify these variables during the injury training process, as well as the maximum speed of the player. It should be noted that an important aspect of the study was the recovery of distance values at different pre-injury speeds, allowing optimal adaptation to group training and competition. One of the most interesting aspects of the on-field reconditioning program was to adapt the tissue to the different speeds that we applied during the sessions [18] in order to get the tissue and the injured athlete closer to the conditional demands of training and competition. The aim was to increase the volume of HSR and VHSR through specific tasks, with a predominance of uncertainty situations with balls and predominantly through speed and speed–endurance conditioning exercises [12,16]. One of the aspects to take into account during this phase was the development of technical–tactical tasks with low levels of uncertainty, following the ‘control-chaos continuum’ model defined by Taberner et al. (2019), considering important specific factors such as positional-specific conditioning, progressive exposure to HSR volume and maximal speed running under increasingly chaotic conditions. The qualitative aspects of movement in competition were also considered, as well as progressive variable, spontaneous and unanticipated movements reflecting the unpredictable nature of sport [17]. In addition, we anticipate increased training loads from the team and, therefore, aim to return the player to his cumulative training HSR volume to adequately prepare the player for these demands [12].

Another limitation that should be highlighted was the absence of objective strength tests, such as measuring the maximum voluntary isometric contraction test (MVICT) capacity through tools that provide us with this information. It should be noted that, despite being in a professional club, resources in certain areas were limited, and some decisions of the medical and technical staff were beyond the control of the investigators. However, clinical assessment through ultrasound imaging, functional and performance criteria, as well as the importance of the multidisciplinary team, were crucial to the process. The follow-up was 6 weeks due to the injury occurring at the end of the season; this can be seen as a limitation, although it should be noted that RTPs will always be conditional on technical decisions.

## 4. Conclusions

Early intervention with PNE and a specific training program for adductor longus injury seem to allow a safe return to competition. In addition, the use of GPS during the training process allowed us to achieve a performance profile close to that of the competition. In our experience, return-to-play processes must contain clear phases and criteria, a philosophy that is not limited to static time-based markers but uses objective performance-related criteria in order to succeed in the injury process.

## Data Availability

The original contributions presented in the study are included in the article. Further inquiries can be directed to the authors.
